# Lipid-based liquid crystalline materials in electrochemical sensing and nanocarrier technology

**DOI:** 10.1007/s00604-023-05727-w

**Published:** 2023-04-18

**Authors:** Martina Zatloukalova, Lukasz Poltorak, Renata Bilewicz, Jan Vacek

**Affiliations:** 1 Department of Medical Chemistry and Biochemistry, Faculty of Medicine and Dentistry, Palacky University, Hnevotinska 3, 775 15 Olomouc, Czech Republic; 2 Electrochemistry@Soft Interfaces Team, Department of Inorganic and Analytical Chemistry, Faculty of Chemistry, University of Lodz, Tamka 12, 91-403 Lodz, Poland; 3 Faculty of Chemistry, University of Warsaw, Pasteura 1, 02-093 Warsaw, Poland

**Keywords:** Lipidic nanoparticles, Lipidic cubic phase, Cubosome, Hexosome, Biologically active compounds

## Abstract

Some biologically active substances are unstable and poorly soluble in aqueous media, at the same time exhibiting low bioavailability. The incorporation of these biologically active compounds into the structure of a lipid-based lyotropic liquid crystalline phase or nanoparticles can increase or improve their stability and transport properties, subsequent bioavailability, and applicability in general. The aim of this short overview is (1) to clarify the principle of self-assembly of lipidic amphiphilic molecules in an aqueous environment and (2) to present lipidic bicontinuous cubic and hexagonal phases and their current biosensing (with a focus on electrochemical protocols) and biomedical applications.

**Graphical Abstract:**

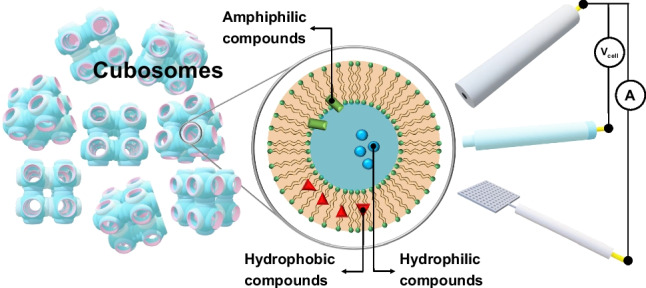

## Introduction

Surfactants and amphiphilic substances (lipids) are not only basic biomolecules of the human body, but they are also one of the main components of pharmaceutical, cosmetic, and food industry products. As a class of biomolecules, they are a source of energy, and play a number of vital functions, such as cell differentiation, signal transduction, organ protection and barrier functions, and the synthesis of essential biomolecules including hormones and bile acids [1]. Lipids are amphiphilic substances that contain hydrophilic and hydrophobic moieties in their molecular structure. The non-polar bonds of the hydrophobic part of the molecule are the reason for their limited solubility in aqueous media. The solubility of lipids and other amphiphilic molecules is characterized by a critical micellar concentration (CMC) [2]. When the CMC of individual monomers is exceeded, higher lipidic structures such as micelles, liposomes, lipidic nanoparticles, and lipidic lyotropic liquid crystals are formed in an aqueous medium [3].

At the turn of the nineteenth and twentieth centuries, the Czech-Austrian biologist Reinitzer [4] discovered that the cholesteryl ester of benzoic acid passes into a liquid state at a temperature of 145 °C; up to 179 °C, it has a milky coloration; and from 179 °C upwards, it is a clear liquid. His pilot studies were followed by the physicist Lehmann [4], who called these states of mater/substances “mesophases” (later, liquid crystals). Liquid crystals are a transition between liquid and solid crystalline states, and thus, they have the properties of both solid substances (ordered and oriented molecules) and liquids (mobility, fluidity). Liquid crystals can be obtained by dissolving a solid substance in a solvent (lyotropic liquid crystals) or by melting (thermotropic liquid crystals). Liquid crystals can also form in an aqueous medium from some lipids [4].

A lipid-based lyotropic liquid crystalline phase is a material that imitates biological membranes. Thus, it represents a suitable matrix for stabilizing hydrophilic, hydrophobic and amphiphilic biologically active compounds, which are often relatively unstable with limited or no solubility in aqueous media. The incorporation of these relatively unstable molecules into carrier media expands the range of possibilities for their stabilization, transport, and controlled (frequently targeted) release. The techniques for nanoencapsulation are constantly evolving, providing new options for the preparation and application of targeted formulations [5].

Over the last few decades, there has been a focus on developing new ways of targeted drug administration. Ideally, the transport and delivery system of such drugs should meet several prerequisites. It should have a high capacity for incorporating substances, be stable and biocompatible, allow for the controlled release of substances, and be targeted at the site of action [6]. Current conventional forms of medication, including controlled dosage release, do not meet all of these preconditions. On the other hand, a number of nanoforms (including polymer nanoparticles and nanocapsules, liposomes, solid lipidic nanoparticles, phytosomes, nanoemulsions, and others) have significant advantages, including increased solubility and bioavailability, stability, and improved tissue distribution. Nanoforms with incorporated biologically active substances thus have the potential to increase the bioavailability and stability parameters of drugs and biologically and pharmacologically active substances in general. Moreover, the combination of drugs can be delivered in one cubosome or hexosome, lipidic cubic phase (LCP)-based nanoforms, and by adding special groups to the nanoform, addressable delivery vehicles can be achieved.

In addition to drug delivery systems, the use of lipid membranes and functional layers in biosensors is frequently discussed today [7, 8]. Biosensors based on lipid membranes make it possible to investigate the properties of membranes and membrane proteins, but also investigate the influence of biologically active substances on their function and stability. The stability of lipid membrane-based biosensors limits their practical use [9]. There are several approaches to achieving stable lipid membranes on electrode surfaces: solid supported lipid bilayers, polymer cushioned bilayers, hybrid lipid bilayers or multilayers, the classical black lipid membrane, and others [10]. The above-mentioned instability could also be overcome by using LCP-based layers or nanoforms. In addition, the multifunctionality of cubosomes/hexosomes could open up new possibilities, as was recently reported with loading enantiomeric ligands, which resulted in the preparation of chiral LCP systems [11, 12].

The aim of this review is to briefly describe the preparation, classification and application of lipid mesophases, the incorporation of proteins into LCP, and the formation and applications of lipid nanoparticles (cubosomes and hexosomes). The future directions of this research are also highlighted, including a strategy towards lipid-based object detection at polarized liquid–liquid interfaces.

## Lipidic mesophase

**Fig. 1:**
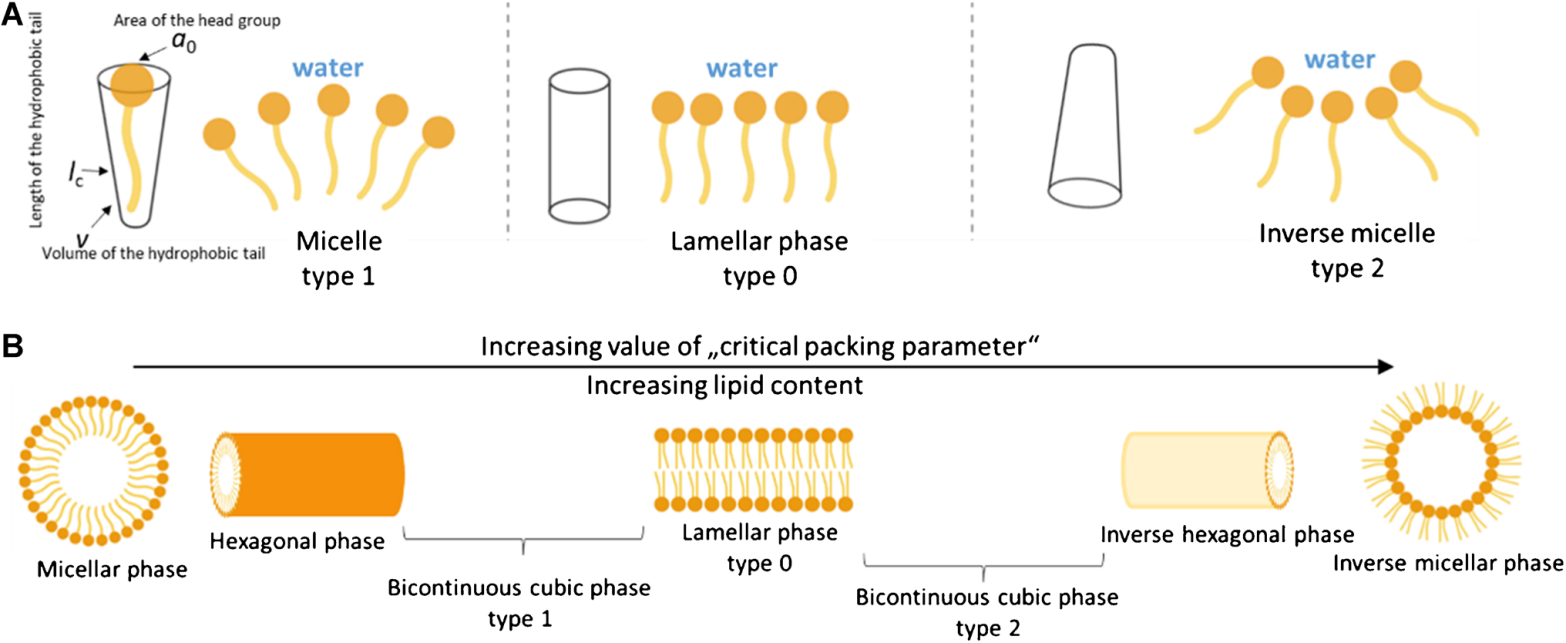
**A** Shape and **B** type of self-assembly systems of amphiphilic molecules (schematically) after contact with water. Modified as described in refs [13, 14]

The spontaneous arrangement of amphiphilic molecules into organized structures is one of the features of many biological systems such as the cell plasma membrane, endoplasmic reticulum, Golgi apparatus, and the densely convoluted mitochondrial membrane. This is the inspiration for the development of new biomimetic materials [5]. The goal of self-assembled formations is to achieve an energy-advantageous state. One of the main drivers of the formation of these supramolecular structures is the hydrophobic effect. However, there are many factors that determine and influence the structure and stability of individual self-assembled formations. In this regard, a decisive role is played by the concentration and shape of the amphiphile. If the concentration of the amphiphile is equal to or higher than the CMC, and the temperature is higher than the critical micellar temperature (also known as the Krafft temperature), the formation of micelles occurs. Amphiphiles differ mainly in the size and shape of the hydrophilic and hydrophobic molecular moieties, which is reflected in their spatial arrangement (Fig. 1).

Surfactants tend to form self-assembled cone-shaped type 1 structures, while molecules that have a smaller polar part, such as lipids, tend to form inverse micellar phases of type 2. Biological membranes composed of a wide range of molecules of varying shapes form dynamic self-assembled formations, including local planar/lamellar structures [15]. The shape of self-assembled formations can be qualitatively described according to Israelachvili et al. [2]. This theory is based on a dimensionless parameter, the so called critical packing parameter (CPP). The CPP is defined according to the following equation:

$$CPP=\frac{v}{{a}_{0}{l}_{\mathrm{c}}}$$

where *v* is the volume of the hydrophobic tail, *a*_0_ is the area of the head group, and *l*_c_ is the length of the hydrophobic tail. Spherical and cylindrical micelles are formed both in the self-arranged phase of type 1 and in the inverse micellar arrangement of type 2.

**Fig. 2:**
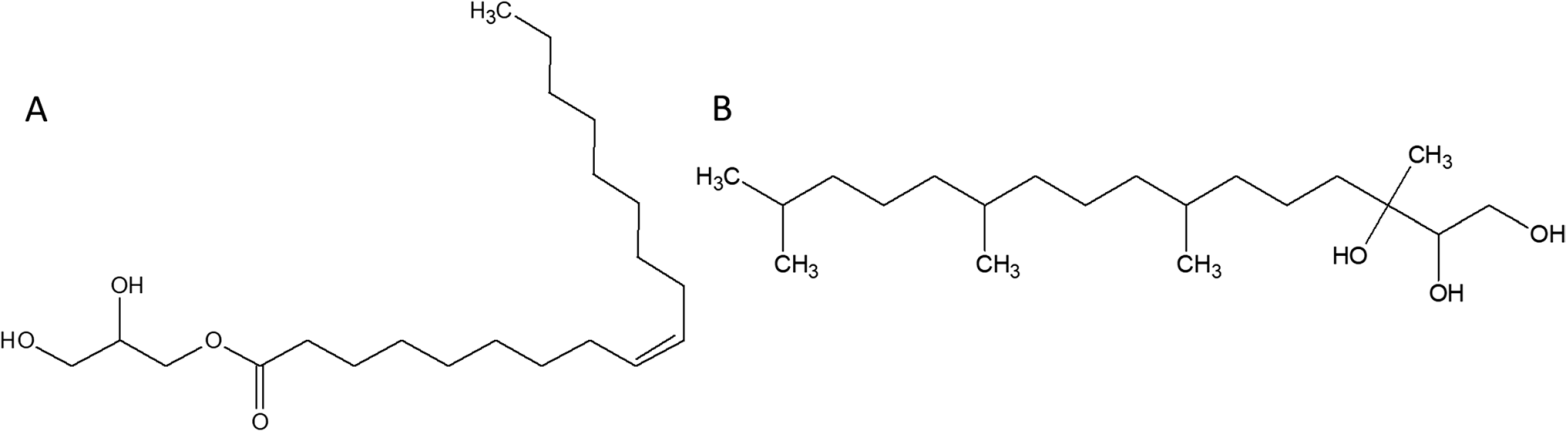
Structure of **A** 1-monoolein and **B** phytantriol

Amphiphiles in an aqueous medium have a spatial arrangement, with the basic lipidic phases designated as lamellar, hexagonal, and bicontinuous cubic phases. With specific components added to the system, other phases can also form [15]. Most common mesophase lipidic structures are based on 1-monoacylglycerol (1-monoolein) and phytantriol, and these are shown in Fig. 2.

**Fig. 3:**
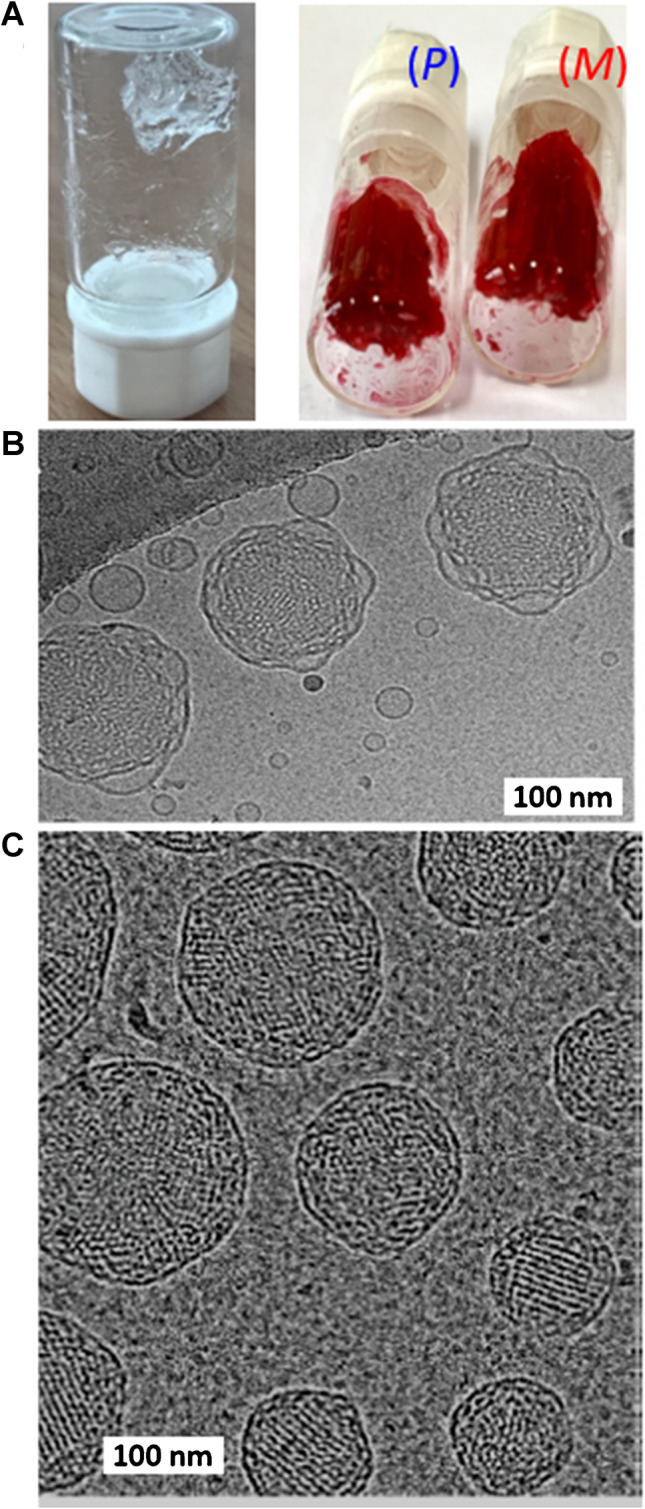
**A** Pure lipidic cubic phase (left) and lipidic cubic phase loaded with *P* and *M* enantiomers of flavo[7]helicene (right); for more details, see ref. [12]. Cryo-TEM images of **B** 1-monoolein- and **C** phytantriol-based cubosomes as described in more detail in refs [19, 20]

**Fig. 4:**
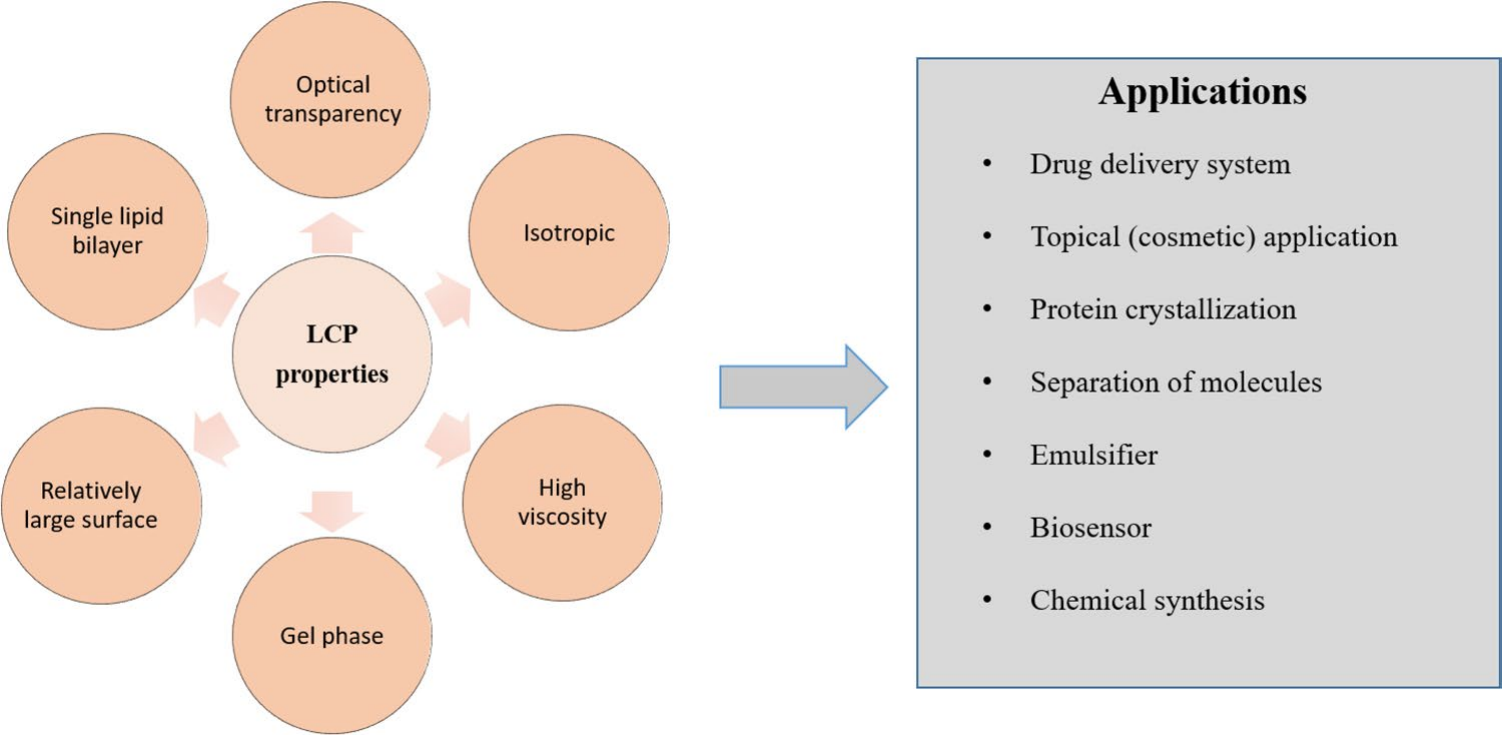
Scheme depicting properties and applications of LCP (lipidic cubic phase). Based on recent reviews in the field: drug delivery [21], crystallization [22, 23], biosensors [10], and a very recent review [24]

1-Monoolein (1-(cis-9-octadecenoyl)-*rac*-glycerol, MO) is a clear viscous substance with a characteristic odor [16]. MO is insoluble in water, but dissolves well in oil and lower hydrocarbons such as chloroform. In particular, due to its high solubility in oil, MO is used as a food emulsifying agent. It is non-toxic, biodegradable, and biocompatible [13]. The properties of amphiphilic molecules are crucial for the final composition and structure of the individual phases, of which a bicontinuous cubic phase in particular has a high degree of spatial organization [17, 18]. An important condition for the reproducibility of the formation of these spatially ordered phases is the precise definition of the default components used and their ratio. A lipidic cubic phase (LCP) is one of the many liquid crystalline phases that spontaneously form when lipids are mixed with water under appropriately selected conditions: water-to-lipid ratio and temperature range. A schematic presentation of the procedure used for the preparation of LCP is shown in Fig. 3A. LCP is a thermodynamically stable, self-assembled lipidic phase with unique properties and structure. The cubic phase consists of two continuous phases, one of which is a lipid bilayer, and the other is formed of aqueous channels. The LCP is characterized by a crystallographic spatial arrangement with *Im3m* (primitive), *Pn3m* (double diamond), or *Ia3d* (gyroid) symmetry [13]. In the *Im3m* phase, the water channels meet in six-way junctions at an angle of 90°. The *Pn3m* phase is characterized by a four-way crossing of water channels at an angle of 109.5°. Cryo-TEM images of LCP nanoparticles based on MO and phytantriol, both with *Pn3m* symmetry, are shown in Fig. 3B, C. Three-way junctions intersecting at an angle of 120° are typical for the *Ia3d* phase [13]. The cubic phase is an attractive tool for a number of different biosensing, biomedicine, and food industry applications (Fig. 4).

## Integration of proteins into the lipidic cubic phase

The cubic phase has been used for more than two decades as a matrix for the crystallization of integral membrane proteins. Landau and Rosenbusch were the first to use a LCP for the crystallization of a membrane protein in 1996 [22]. Today, there are more than 700 proteins in the protein databank (PDB) that have been crystallized in a LCP (crystallization *in meso*) [25–31]. These include a number of enzymes, transporters [32], channels, receptors [33], and structural proteins [34]. Protein crystallization in a LCP made it possible to elucidate the structures of several microbial rhodopsins and receptors associated with G-proteins. Biomimetic membranes such as LCPs provide proteins with a more natural membrane environment, as opposed to the artificially created environment associated with the presence of detergents [27, 35, 36]. An example of the use of a LCP for the crystallization of polar, water-soluble proteins is lysozyme. Unlike membrane bacteriorhodopsin [37, 38], lysozyme crystallizes independently of the lipidic phase type [23]. LCP technologies for structural studies of membrane proteins such as bacteriorhodopsin or gramicidin were reviewed by Cherezov [39]. The most common cubicon method for the incorporation of membrane proteins was described by Ma et al. [40]. The organization of proton pumps, lipids, and water in cubic mesophase crystals was discussed by Belrhali et al. [41].

The functional properties of the proteins are retained in the lipidic environment, as shown in several reports [42]. They can therefore be used as active components of lipid-liquid-crystalline films, e.g., deposited on solid surfaces. A viscous, stable three-dimensional lipid bilayer with incorporated membrane proteins/redox probes is easily applied to an electrode surface [43]. Less recent applications in sensing are presented in refs [10, 44]. Fructose dehydrogenase (FDH) was reconstituted in a monoolein cubic mesophase and used for the electrochemical determination of fructose [45]. Mezzenga et al. confirmed in a spectrophotometric study that *in meso*-immobilized FDH has improved stability compared to other matrices or solutions [46].

In a recent study, we reconstituted and showed the activity of the Na^+^/K^+^-ATPase (sodium–potassium pump, NKA) in a MO-based LCP for the first time [47]. The reconstitution and chloride transport of the chloride transporter protein EcClC in the LCP were described by Speziale et al. [48] and by ourselves [49], and the efficiency of chloride transport was studied using inhibitors and activators of the protein. Active gating was demonstrated for the glucose transporter protein [50]. Lipidic liquid-crystalline mesophases are also used for protein biochip development [51].

Polar analytes reside in the aqueous channels of lipidic mesophases, providing free access to the electrode surface and proteins. These substances easily communicate with the electrode directly or *via* an electroactive probe [10]. In 1994, Razumas et al. [52] were the first to report on the preparation of LCP biosensors. The first biosensors based on enzymes incorporated into a LCP were designed to determine glucose, lactate, urea, and creatinine. Current responses dependent on H_2_O_2_ oxidation were detected amperometrically. Table 1 summarizes published electrochemical enzyme biosensors based on a LCP.

**Table 1:** Electrochemical enzyme biosensors based on LCP

Enzyme/protein	Method	Electrode	Ref.
Glucose oxidase, ceruloplasmin	Amperometry	Platinum disk	[53]
Hemoglobin	CV, amperometry	Glassy carbon electrode	[54]
Glucose oxidase, lactate oxidase, urease, creatinine deiminase	Amperometry, potentiometry	Platinum electrode, pH electrode	[52]
Glucose oxidase, pyranose oxidase, laccase	CV	Glassy carbon electrode	[55]
Glucose oxidase	CV	Carbon electrode	[56]
Na^+^/K^+^-ATPase	SWV	Glassy carbon electrode	[47]
Ethanol dehydrogenase	CV, DPV	Glassy carbon electrode	[57]
Laccase ^#^	Chronoamperometry, CV, AC impedance	Modified glassy carbon electrode	[58]
Cellobiose dehydrogenase	CV, DPV	Modified glassy carbon electrode	[59]
Cholesterol oxidase	CV	Glassy carbon electrode, gold electrode	[60]
Bilirubin oxidase	CV	Carbon rotating disk electrode	[61]

*CV*, cyclic voltammetry; *SWV*, square wave voltammetry; *DPV*, differential pulse voltammetry; ^#^discussed with Rowinski et al

These studies show that proteins can be incorporated into the lipidic phase at relatively high concentrations, and they readily interact with the surface of the electrode. However, what should be taken into account is the influence of physico-chemical factors such as the ratio of water to lipid in the mesophase, temperature, pressure, lipid composition, and the presence of other substances. These factors could influence the phase transition of the LCP, lead to its destabilization, and promote the subsequent release of incorporated enzymes due to the transition of the LCP to a hexagonal or lamellar phase.

In addition to protein crystallization and biosensor construction, a LCP can also be used as a matrix for research into the stability, activity, and interaction of proteins with ligands [47, 62, 63]. One example is a stability study of the above-mentioned NKA. NKA carries sodium and potassium ions against their concentration gradient using energy from ATP hydrolysis. In this experiment, after 14 days, the NKA activity in the LCP was still 60% of its maximum activity, while it was no longer active in the parallel experiment incubated in an aqueous environment [47]. A model example of research on the redox behavior of proteins incorporated into a LCP are studies conducted using cytochrome *c* [63–65]. The interaction of cytochrome *c* with a LCP mimicking the environment of the inner mitochondrial membrane was investigated using FTIR spectroscopy, differential scanning calorimetry, and electrochemical techniques. The diffusion coefficient of cytochrome *c* was determined using electrochemical methods, and it showed very limited protein mobility in the lipid environment; more details can be found in a relatively recent review [36].

## Electroactive probes and lipidic cubic phase

Barauskas et al. [66] prepared an electrochemically active cubic phase containing various types of amphiphilic substances (ferrocenes). A fold decrease in the diffusion coefficient of amphiphiles incorporated into the LCP was observed compared to the diffusion coefficient determined in acetonitrile solutions. Rowinski et al. [43] investigated the behavior of hydrophilic probes in a cubic phase using cyclic voltammetry, differential pulse voltammetry, and chronocoulometry. The diffusion coefficients for [Ru(NH_3_)_6_]^3+^ and benzoquinone in the LCP were found to be lower and in accordance with the diffusion coefficient determined in solution. Similarly, Kostela et al. [67] determined the diffusion coefficient of hydrophilic, hydrophobic, and amphiphilic electroactive probes in a LCP. The diffusion coefficients for these electrochemically active species were also determined in a hexagonal phase using electrochemical and impedance methods [68]. In-depth experimental and theoretical studies of diffusion in a LCP have been reported more recently [69, 70].

## Lipidic nanoparticles, general aspects

**Fig. 5:**
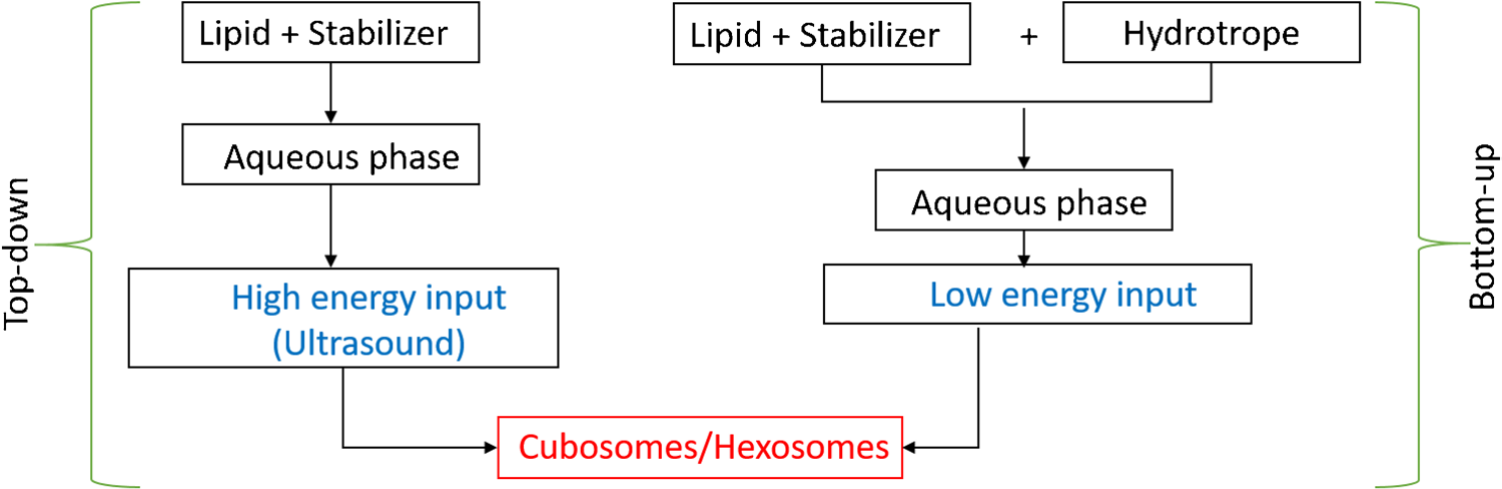
Schematic representation of “top-down” and “bottom-up” general approaches for preparation of cubosomes and hexosomes

New lipid-based nanocarriers are constantly being sought and developed for the design of biosensors and the development of transport and application systems. One of the key advantages of using lipidic nanoparticles is the effective solubilization of poorly water-soluble substances, as demonstrated, for example, with curcumin [71] and quercetin [72]. The most explored lyotropic nanostructure carriers include cubosomes and hexosomes, which are defined as colloidal nanoparticles with internal bicontinuous cubic and hexagonal structures. This group of colloidal dispersions is called ISAsomes (internally self-assembled “somes” or particles). In addition to cubosomes and hexosomes, ISAsomes include micellar cubosomes [73]. The first mention of the existence of cubosomes dates back to the 1980s, when Larsson discovered that the dispersion of LCPs produces submicron particles with an identical internal arrangement to the parent cubic structure [74]. Cubosomes are highly stable nanoparticles formed from a lipidic cubic phase and stabilized by the outer layer. Compared to liposomes, cubosomes and hexosomes provide a significantly larger surface area (up to 400 m^2^/g) for the incorporation of membrane proteins and small hydrophilic or hydrophobic molecules [75]. In general, there are two main approaches to the preparation of cubosomes, the “top-down” and “bottom-up” approach, both of which require the use of a suitable stabilizer (Fig. 5).

The “top-down” method, the most widely used and oldest technique used for the preparation of cubosomes [16], involves two main steps. First, the LCP is prepared, which is then homogenized/sonicated using high-energy pulses. Cubosomes prepared by the “top-down” method are stable against aggregation for up to 1 year. The disadvantage of this method is however the use of high-energy pulses, which can affect the activity of incorporated biologically active substances sensitive to elevated temperature [75].

The second method, commonly called the “bottom-up” method, involves the dispersion of a mixture containing lipid, stabilizer, hydrotrope, and an excess of water (Fig. 5) [76]. The hydrotrope is a key factor in this process and helps to solubilize lipids [77]. The most frequently used hydrotropes are urea, sodium alginate, sodium benzoate, or ethanol. The advantage of this approach is the use of less energy, so that it can also be applied for the preparation of cubosomes with thermounstable substances such as peptides and proteins [16]. The same preparation procedure can be used for hexosomes [78]. The LCP phases dispersed into nanoparticles used as drug nanocarriers were recently reviewed by Angelova et al. [79], Murgia et al. [80], and Tenchov et al. [81].

To prevent the aggregation of ISAsomes, stabilizing components have to be added during the synthetic procedure. Over the last decade, various types of stabilizers have been proposed for the preparation of cubosomes and hexosomes. The most common and effective of these surfactants is Poloxamer 407, which is commercially available under the name Pluronic F127 [82, 83]. In addition to Pluronic F127, other stabilizing agents have been proposed, including various co-polymers such as F128, F108 [84, 85], PEGylated lipids, or β-casein [86]. The choice of stabilizer for the preparation of ISAsomes is critical, as stabilizers can also affect the internal lipidic nanostructure and thus the affinity towards the cargo [87].

Liquid crystalline phases and their corresponding aqueous dispersions are characterized primarily by two techniques, namely, SAXS (small-angle X-ray scattering) and SANS (small-angle neutron scattering) [88]. Studies are mainly focused on describing the influence of physico-chemical factors on the structural properties of nanoparticles, including lipid composition, temperature, pH, pressure, and the effect of target substance incorporation. In addition to SAXS and SANS, cryo-TEM [89] and AFM [90] are also used for morphological characterization. Dynamic light scattering (DLS) is frequently employed for monitoring particle size. To demonstrate the use of nanocarriers for biosensor construction, we present work based on phytantriol-based cubosomes stabilized with F127. The solid gold surface of the sensor was modified through biotinylated lipids that were part of the cubosomes. The second set of cubosomes enriched with glycolipid (GM1) was then applied to the modified surface, and this led to the specific binding of cholera toxin B from solution [91].

### Applications of cubosomes and hexosomes

These days, we are seeing a growing interest in the use of ISAsomes, especially cubosomes and hexosomes, as nanocarriers for sensing strategies and for drug incorporation, imaging probes, and antimicrobial peptides. Particular attention is paid to the solubilization and stabilization of biologically active substances, the influence of lipid composition, and the type and concentration of the stabilizer on the structural and morphological properties of these nanoforms. On the other hand, there have been few studies on the release of substances from cubosomes/hexosomes and factors affecting the stability of these structures, especially in a living organism [92, 93]. There is increasing interest in the influence of incorporated substances or cubosomes/hexosomes themselves on cell signaling pathways. The release of doxorubicin from the cubic lipidic phase depending on the change in pH (with respect to differences in pH of tumors *vs.* non-malignant tissue) was studied using electrochemical methods [94]. The bioavailability (cell uptake kinetics) and cytotoxicity of cubosomes have been investigated mainly *in vitro* on cell lines [83, 95, 96]. Cubosomes and hexosomes are beginning to be applied in theranostics. DLS, SAXS, and cryo-TEM methods have shown that hexosomes are able to incorporate both a fluorescent probe and the anticancer drug camptothecin into their structure. Fluorescence microscopy has shown that the HeLa cell line is able to accumulate modified hexosomes. For fluorescence microscopy, non-toxic concentrations of modified hexosomes were used [97]. Cytryniak et al. [19, 98] were the first to demonstrate the use of cubosomes in radiotherapy in combination with chemotherapy. MO-based cubosomes were modified by incorporating the anticancer drug doxorubicin and a commonly used radionuclide. The cytotoxicity of the modified cubosomes was tested on HeLa cells. Cubosomes modified with doxorubicin and the radionucleotide were shown to be more toxic than cubosomes alone or cubosomes with an incorporated chemotherapeutic/radionucleotide. The combination of cryo-TEM and SAXS methods was used to examine the impact of blood plasma on the size, structural, and morphological properties of cubosomes over time [99]. However, few other studies have focused on the stability of lipidic cubic and hexagonal nanoparticles in the bloodstream, structural transformations of cubosomes/hexosomes after contact with cell membranes, blood cells, or proteins, or their cell uptake. The structural arrangement of bicontinuous cubic and hexagonal phases allows for a gradual release of incorporated substances. Bicontinuous cubic nanostructures have also been shown to have mucoadhesive properties [100, 101]. The use of these 3D-nanolipidic structures was mainly intended for the oral, subcutaneous, transdermal, and periodontal administration of biologically active substances. Lipidic liquid crystalline phases are highly viscous and therefore have limited use as intravenous nanocarriers. Cubosome/hexosome suspensions are much less viscous and therefore much more convenient for drug delivery. The most important aspect of these drug delivery studies is related to the degradation of lipidic nanoparticles in the biological environment as a result of interactions with, e.g., enzymes and macrophages or other species present in the biological medium [102]. An interesting future direction of research could also be the use of lipidic vehicles for the incorporation of biologically active fatty acids and lipids [103]. A more detailed overview of the use of cubosomal/hexosomal lipidic structures based on MO or phytantriol for targeted drug delivery systems is provided in the following publications [5, 16, 73, 75, 78, 104].

## Future directions: lipid-based objects and polarized liquid–liquid interfaces

**Fig. 6:**
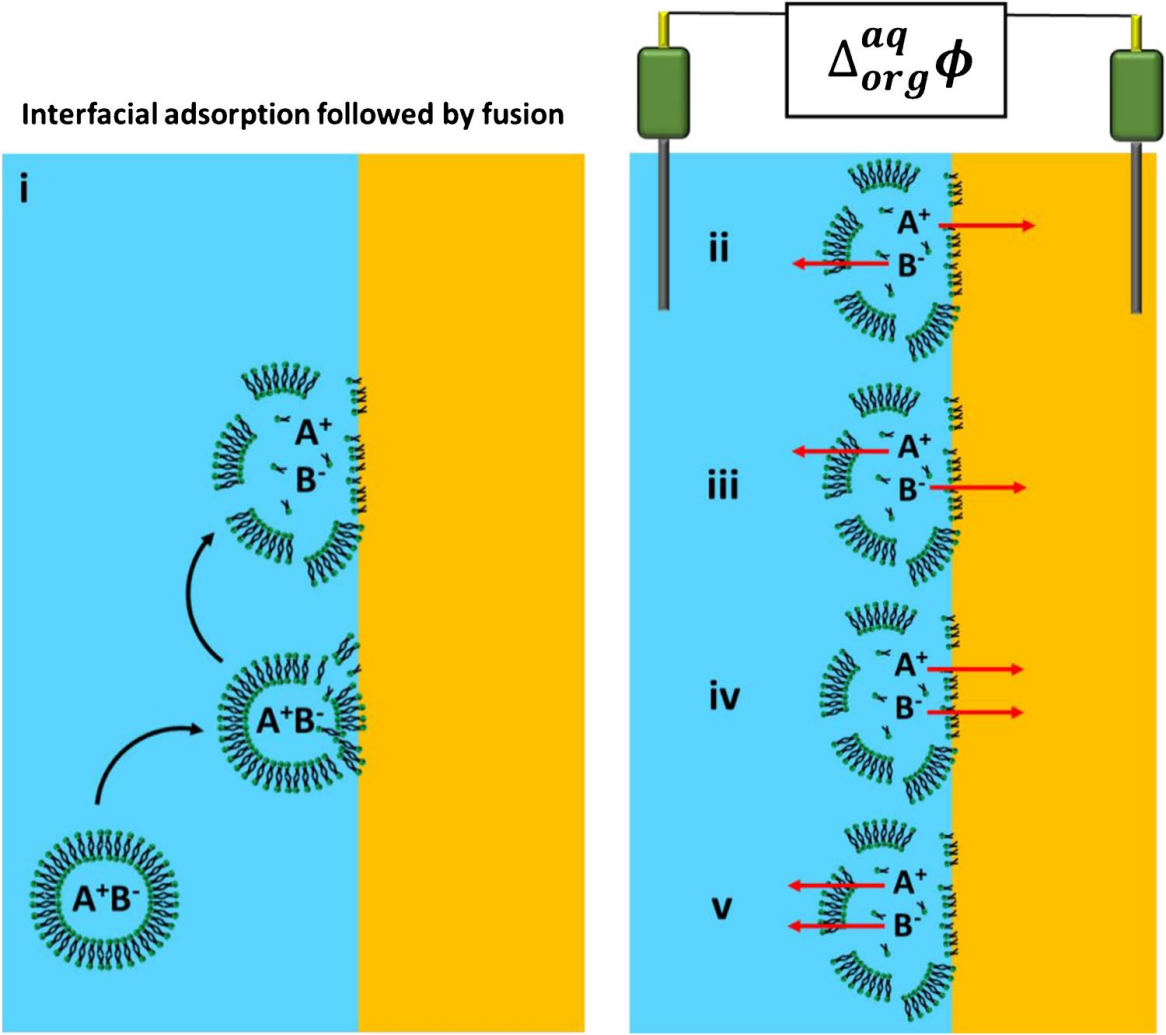
**i** Schematic representation of concept assuming that liposome approaching liquid–liquid interface, upon interfacial adsorption, will fuse and release its cargo. **ii**–**v** are possible lipid-based object cargo—salt dissociated into ions/charged chemical species—interfacial ion transfer reactions

Electrochemical studies of lipid-based objects, including lipidic nanoparticles and micelles, should go beyond configurations involving the utilization of solid electrodes [105]. We postulate that the concept introduced by Laborda et al. [106] and further studied by a few other groups [107–109], which describes the single fusion events of an emulsion droplet hitting a polarized liquid–liquid interface, can be further developed and adapted to study the interactions of lipid-based objects with soft interfaces. This aspect is visualized in Fig. 6. One can imagine that the lipidic object, e.g., a liposome, when placed next to the electrified liquid–liquid interface (either bare or modified with a lipid monolayer [110]), can undergo fusion and the release of its cargo. When the Galvani potential difference (${\Delta }_{org}^{aq}\phi$) is fixed to a value higher than ${\Delta }_{org}^{aq}{\phi }_{{A}^{+}}$ and ${\Delta }_{org}^{aq}{\phi }_{{B}^{-}}$ (see Fig. 6, ii), it is expected that cationic species will transfer to the organic phase, whereas anions will remain in the aqueous phase. When ${\Delta }_{org}^{aq}\phi$ is lower than ${\Delta }_{org}^{aq}{\phi }_{{A}^{+}}$ and ${\Delta }_{org}^{aq}{\phi }_{{B}^{-}}$ (see Fig. 6, iii), it is only the anion that should transfer to the organic phase; cations will stay in the aqueous phase. Both species are expected to undergo interfacial ion transfer when ${\Delta }_{org}^{aq}\phi$ is higher than ${\Delta }_{org}^{aq}{\phi }_{{A}^{+}}$ and lower than ${\Delta }_{org}^{aq}{\phi }_{{B}^{-}}$ (see Fig. 6, iv). The opposite situation, i.e., when ${\Delta }_{org}^{aq}\phi$ is lower than ${\Delta }_{org}^{aq}{\phi }_{{A}^{+}}$ and higher than ${\Delta }_{org}^{aq}{\phi }_{{B}^{-}}$, restricts partitioning. In this way, a full range of electrochemical techniques can be applied to either study or control the ionic partitioning of the molecular cargo carried by lipid-based objects. Moreover, we believe that properly designed experiments, in which lipidic objects carry an ionic cargo, can be used to follow the impacts of the lipid-based objects with a liquid–liquid interface-, fusion efficiency-, and potential-dependent adsorption of the lipid objects to a polarized junction. A polarized junction is a biointerface that mimics a real cell membrane with established cell potential. The impacts are detected in the form of changes in the electric properties of the interfacial region that follow the ionic currents originating from the interfacial charge transfer reaction involving the encapsulated molecular cargo.

## Conclusions

Highly organized 3D-lipidic architectures can be used as matrices for the crystallization of integral membrane proteins or in the development of new lipidic nanoforms for both analytical applications and the targeted transport and stabilization of biologically active molecules. Knowledge of the optimal internal lipid arrangement and selection of suitable agents for the stabilization of nanocarriers is essential for designing a specific usable lipidic matrix. Although we understand the unique properties of cubosomes and hexosomes, there are still a number of questions about the fate of nanocarriers after their *in vivo* administration. Similarly, there is limited knowledge in the field of cell testing, including a deeper understanding of the mechanism of interaction with cell membranes and receptors, the actual entry into cells, and the release of biologically active substances from nanocarriers. As for sensing and the development of LCP-modified electrodes or microarray detection surfaces, fundamental research of interfacial behavior would be an important direction in the future.

## Abbreviations

*AFM*: Atomic force microscopy
*CMC*: Critical micellar concentration
*CPP*: Critical packing parameter
*Cryo-TEM*: Cryo-transmission electron microscopy
*DLS*: Dynamic light scattering
*FDH*: Fructose dehydrogenase
*ISAsomes*: Internally self-assembled “somes” or particles
*LCP*: Lipidic cubic phase
*MO*: 1-Monoolein
*NKA*: Na^+^/K^+^-ATPase
*PDB*: Protein databank
*SANS*: Small-angle neutron scattering
*SAXS*: Small-angle X-ray scattering
